# P2Y2 Receptor and EGFR Cooperate to Promote Prostate Cancer Cell Invasion via ERK1/2 Pathway

**DOI:** 10.1371/journal.pone.0133165

**Published:** 2015-07-16

**Authors:** Wei-Hua Li, Ying Qiu, Hong-Quan Zhang, Xin-Xia Tian, Wei-Gang Fang

**Affiliations:** 1 Key Laboratory of Carcinogenesis and Translational Research, Ministry of Education, Peking University Health Science Center, Beijing, 100191, China; 2 Department of Pathology, Peking University Health Science Center, Beijing, 100191, China; 3 Department of Anatomy, Histology and Embryology, Peking University Health Science Center, Beijing, 100191, China; University of Central Florida, UNITED STATES

## Abstract

As one member of G protein-coupled P2Y receptors, P2Y2 receptor can be equally activated by extracellular ATP and UTP. Our previous studies have proved that activation of P2Y2 receptor by extracellular ATP could promote prostate cancer cell invasion and metastasis *in vitro* and *in vivo* via regulating the expressions of some epithelial-mesenchymal transition/invasion-related genes (including IL-8, E-cadherin, Snail and Claudin-1), and the most significant change in expression of IL-8 was observed after P2Y2 receptor activation. However, the signaling pathway downstream of P2Y2 receptor and the role of IL-8 in P2Y2-mediated prostate cancer cell invasion remain unclear. Here, we found that extracellular ATP/UTP induced activation of EGFR and ERK1/2. After knockdown of P2Y2 receptor, the ATP -stimulated phosphorylation of EGFR and ERK1/2 was significantly suppressed. Further experiments showed that inactivation of EGFR and ERK1/2 attenuated ATP-induced invasion and migration, and suppressed ATP-mediated IL-8 production. In addition, knockdown of IL-8 inhibited ATP-mediated invasion and migration of prostate cancer cells. These findings suggest that P2Y2 receptor and EGFR cooperate to upregulate IL-8 production via ERK1/2 pathway, thereby promoting prostate cancer cell invasion and migration. Thus blocking of the P2Y2-EGFR-ERK1/2 pathway may provide effective therapeutic interventions for prostate cancer.

## Introduction

Prostate cancer is one of the most common malignancies in human male population [1]. Most deaths related to prostate cancer are due to invasion and metastasis. Cell invasion and metastasis are complex processes that are regulated by multiple signaling pathways such as MAPK, Wnt and Notch pathways. Activation of these pathways is mainly dependent on interactions between receptors and extracellular signaling molecules [2].

Extracellular adenosine 5’-triphosphate (ATP) is an important signaling molecule in tissue microenvironment, which mediates various biological functions via activation of P2 receptors [3]. Two subfamilies of P2 receptors have be_α_ in mammalian cells. One is P2X family of ligand-gated ion channel receptors (P2X1-7), and the other is P2Y family of G protein-coupled receptors (P2Y1, 2, 4, 6, 11, 12, 13, 14) [4]. Our previous study demonstrated that activation of P2Y receptors by ATP enhanced prostate cancer cell invasion [5]. We further found that P2Y2, a preferred receptor for ATP and UTP, contributed to the invasion and metastasis of prostate cancer cells [6]. However, the signaling pathway(s) downstream of P2Y2 receptor, especially in prostate cancer progression, is still not clear.

As a member of the CXC chemokine family, IL-8 expression is low in normal tissue and can be induced by a variety of stimuli such as growth factors and inflammatory cytokines in pathologic conditions [7]. The expression of IL-8 is often elevated in human tumor cells and tissues [8]. It is reported that IL-8 functions as a significant regulatory factor in tumor microenvironment, and plays a crucial role in tumor invasion and metastasis [9]. We previously found that activation of P2Y2 receptor upregulated the expression and secretion of IL-8 [6]. However, the function of IL-8 in P2Y2 receptor-promoted invasion of prostate cancer cells remains unknown. This study aimed to examine the signaling pathway(s) downstream of P2Y2 receptor, and to explore the role of IL-8 in P2Y2 receptor-promoted prostate cancer cell invasion.

## Materials and Methods

### Chemicals and antibodies

ATP (adenosine 5’-triphosphate), UTP (uridine 5’-triphosphate), AG1478 (EGFR inhibitor) and U0126 (MEK1/2 inhibitor) were all purchased from Sigma (St Louis, MO, USA). ATP and UTP were both dissolved in normal saline and used at the concentration of 100 μM. AG1478 was dissolved in DMSO and used at the concentration of 100 nM. U0126 was dissolved in DMSO and used at the concentration of 10 μM. The antibodies of P2Y2 (rabbit polyclonal antibody, sc-20124), β-actin (mouse monoclonal antibody, sc-8432), ERK1/2 (rabbit polyclonal antibody, sc-94) and EGFR (mouse monoclonal antibody, sc-373746) were purchased from Santa Cruz Biotechnology (Santa Cruz, CA, USA). The antibodies of phospho-EGFR (rabbit monoclonal antibody, #8543, Tyr-1068) and phospho-ERK1/2 (rabbit polyclonal antibody, #9101, Thr202/Tyr204) were purchased from Cell Signaling Technology (Danvers, MA, USA).

### Cell lines and culture conditions

Two subclones 1E8 and 2B4 were derived from PC-3M human prostate carcinoma cell line, which was previously purchased from American Type Culture Collection (Manassas, VA, USA). The 1E8 cell line was highly metastatic, whereas the 2B4 cell line was non-metastatic [10]. DU-145 cell line was purchased from American Type Culture Collection (Manassas, VA, USA). All cells were cultured in RPMI 1640 (GIBCO, Grand Island, NY, USA) supplemented with 10% fetal bovine serum, and maintained in a water-saturated atmosphere at 37°C with 5% CO_2_.

### Reverse transcription and real-time PCR

Cells were grown in monolayer with or without ATP treatment. Total RNA was isolated with Trizol reagent (Invitrogen, Carlsbad, CA, USA). Reverse transcription reaction was performed using M-MLV Reverse Transcriptase (Promega, Madison, Wisconsin, USA) to obtain the cDNA, according to the manufacturer’s guidelines. Next, real-time PCR was performed with the primers of IL-8 (sense: 5’-ACTGAGAGTGATTGAGAGTGGAC-3’; antisense: 5’- AACCCTCTGCACCCAGTTTTC-3’) or β-actin (sense: 5’- GGATGCAGAAGGAGATCACTG-3’; antisense: 5’-CGATCCACACGGAGTACTTG-3’). The real-time PCR reaction was performed on an ABI StepOne Real-Time PCR System (Life Technologies, Carlsbad, CA, USA) in triplicates. The 20 μl reaction mixture consisted of 2 μl of cDNA (20 ng/μl), 100 nM of primers and 10 μl of SYBR Green Real-time PCR Master Mix (TOYOBO, Japan) containing AmpliTaq gold DNA polymerase. Samples were first denatured at 95°C for 10 min and then PCR reaction was proceeded for 40 amplification cycles as follows: 15 s at 95°C and 1 min at 60°C. Then a dissociation curve analysis was conducted and melting temperatures (Tm) of the formed PCR amplicons were observed to distinguish the amplified sequences of interest from non-specific ones or primer dimmers. The expression of IL-8 was normalized by β-actin. The 2 –^ΔΔCt^ method was used for relative quantification as described previously [11, 12].

### Cell lysis and western blotting

Cells were rinsed twice with ice-cold PBS and lysed in lysis buffer containing 20 mM Tris-HCl (pH 7.5), 250 mM NaCl, 4 mM EDTA, 0.5% NP-40, 20 mM β-Glycerophosphate, 1 mM NaF with protease inhibitors (Roche, Mannheim, Germany). Protein concentrations were determined using a BCA protein assay kit (Applygen Technologies Inc, Beijing, China). Equal amounts of total protein were loaded and separated by SDS-PAGE gel, and then were transferred to PVDF membranes (Bio-Rad, Hercules, CA, USA). After blocking with 5% BSA at room temperature for 1 h, blots were probed with primary antibodies against P2Y2 (1: 500), β-actin (1: 1000), EGFR (1: 500), ERK1/2 (1: 1000), phospho-EGFR (1: 1000) or phospho-ERK1/2 (1: 1000) at 4 °C overnight. Then the blots were washed with PBS for three times and incubated with secondary antibodies at room temperature for 1 h. The immunoreactive bands were visualized by an enhanced chemiluminescence detection system (Applygen Technologies Inc), and quantified with the software of Quantity One (Bio-Rad).

### siRNA and transfection

Two P2Y2 siRNAs (P2Y2 si#1 and P2Y2 si#2) and a control siRNA (Negative) were used as described previously [6]. Two IL-8 siRNAs (IL-8 si#1 and IL-8 si#2) were used to silence the expression of IL-8. The IL-8 siRNAs were purchased from Invitrogen (Carlsbad, CA, USA) with the sequence as follows:

IL-8 si#1, 5'-GAACTTAGATGTCAGTGCATA-3';

IL-8 si#2, 5'-GCCAAGGAGUGCUAAAGAA-3'.

A fluorescence-labelled siRNA oligonucleotide was used to directly observe siRNA transfection efficiency, and a scramble siRNA oligonucleotide was used as control siRNA (Negative). Cells were seeded into 6-well plates at the density of 1 × 10^4^ cells/well. Twelve hours later, cells were transfected with siRNAs by Lipofectamine 2000 (Invitrogen), according to the manufacturer’s instructions. Six hours later, the medium was replaced with fresh 1640 medium supplemented with 10% FBS. Twenty hours after transfection, cells were split. After an additional 12 h, knockdown efficiency was determined by western blotting, and cells were used for the following experiments.

### Enzyme-linked immunosorbent assay (ELISA)

Cell supernatant was collected to measure the IL-8 protein level using the Quantikine IL-8 ELISA kit (Invitrogen). Samples were assayed in duplicate. Readings were compared with standard curve. Then the cells were lysed in lysis buffer and total protein concentrations were determined by BCA assay. Finally, the relative concentration of IL-8 protein in cell supernatant was determined by normalizing to total protein of the whole cell extract.

### Invasion assay

Invasion assay was performed as described previously [6]. Briefly, cells were harvested and resuspended in RPMI 1640 with 0.1% BSA at a density of 5 × 10^5^ cells/ml. Next, 200 μl cell suspensions with or without ATP treatment was placed in the upper chamber coated with Matrigel (BD, Franklin Lakes, NJ, USA). NIH3T3-conditioned medium was obtained by incubating NIH3T3 cells for 24 h in serum-free 1640 medium and filled in the lower chamber (600 μl) as a chemo-attractant, according to the method described by Albini et al [13]. Cells were allowed to invade for 12 h at 37 °C. After fixed with 4% formaldehyde, cells on the lower surface of the membranes were stained with crystal violet and observed under a microscope at × 200 magnification. The number of invaded cells in seven fields was counted and the mean for each chamber was determined.

### Migration assay

One hundred thousand cells in 100 μl of RPMI 1640 supplemented with 0.1% BSA was placed in the upper chamber. NIH3T3-conditioned medium was obtained by incubating NIH3T3 cells for 24 h in serum-free 1640 medium and filled in the lower chamber (600 μl) as a chemo-attractant. Cells were incubated for 12 h at 37°C with or without ATP treatment. Then cells on the lower surface of the membrane were fixed and stained with crystal violet. The number of migrated cells was counted under a light microscope at × 200 magnification. The average number of migrated cells was determined from seven representative fields.

### Statistical analyses

The quantitative results were presented as the means ± SD of three determinations and data were analyzed with the software package of SPSS 19.0 (SPSS Inc., Chicago, IL, USA). Student’s t test was used to detect whether there was a significant difference between two groups. Nonparametric ANOVA was used when multiple groups were compared. Any P-value of less than 0.05 was considered to be statistically significant.

## Results

### Extracellular ATP or UTP stimulation activates EGFR and ERK1/2

**Fig 1:**
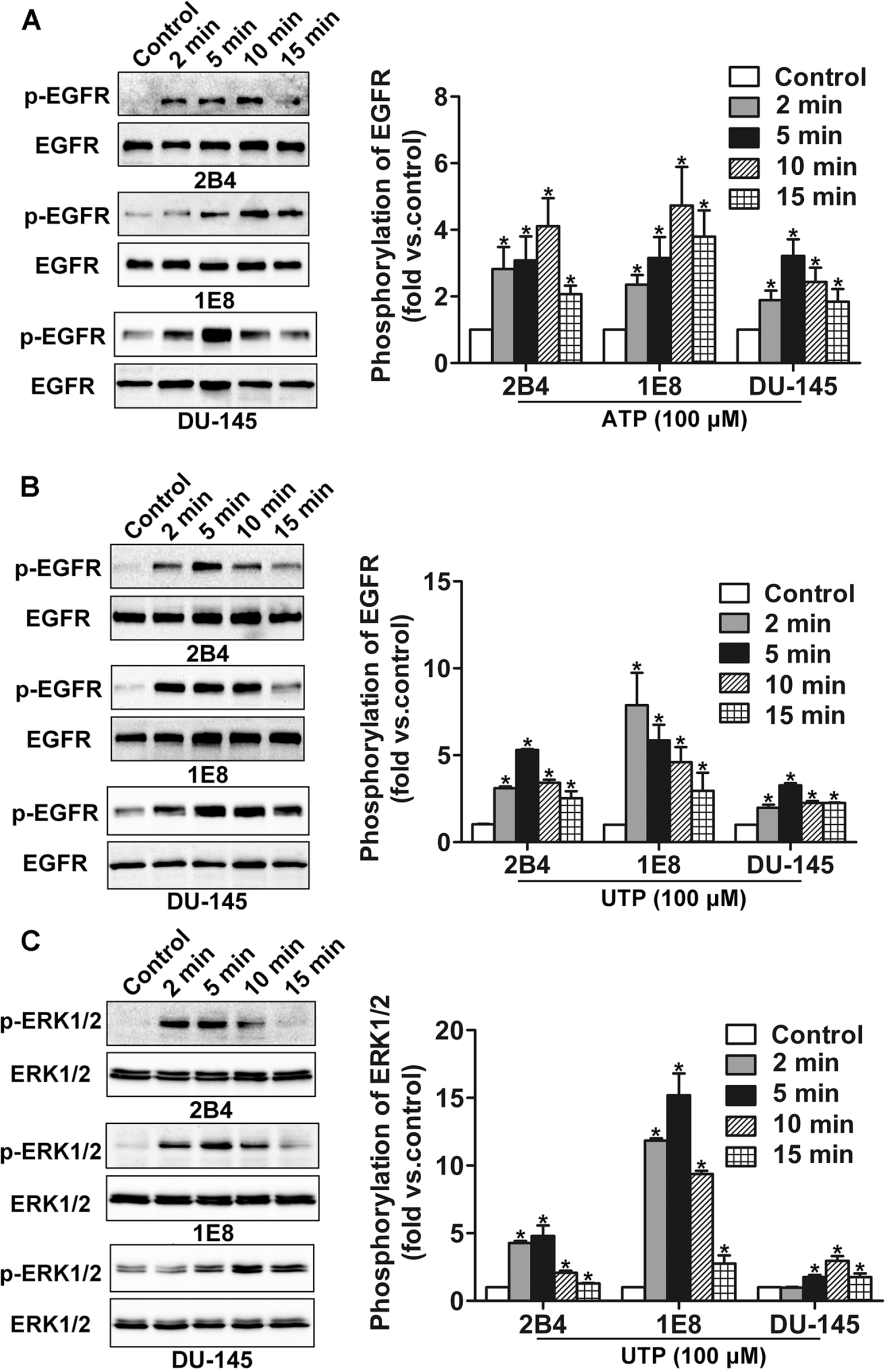
Phosphorylation of EGFR was detected after ATP or UTP treatment. (**A**) After treatment with 100 μM ATP, phosphorylation of EGFR was detected by western blotting. (**B**) After treatment with 100 μM UTP, phosphorylation of EGFR was detected by western blotting. (**C**) After treatment with 100 μM UTP, phosphorylation of ERK1/2 was detected by western blotting. Results were demonstrated by histograms to quantify the expression levels. Data were presented as means ± SD (vertical bars). Three independent experiments were performed. *p<0.05 vs. control cells.

In the present study, after incubation with 100 μM ATP, we detected the phosphorylation of EGFR in 2B4, 1E8 and DU-145 cells by western blotting at 2 min, 5 min, 10 min and 15 min. Our results showed that ATP stimulation resulted in activation of EGFR (Tyr-1068) in prostate cancer cells (Fig 1A). Similar results were observed with UTP treatment (Fig 1B). Our previous study showed that ATP could induce activation of ERK1/2 in prostate cancer 2B4, 1E8 and DU-145 cells [12]. Here, we further found that UTP also stimulated activation of ERK1/2 (Thr202/Tyr204) in 2B4, 1E8 and DU-145 cells (Fig 1C). Among eight P2Y subtype receptors identified in mammalian cells, only P2Y2 receptor can be equally activated by ATP and UTP [14]. Therefore, these results indicate that P2Y2 receptor may be involved in extracellular ATP/UTP-induced activation of EGFR and ERK1/2. The following experiments were only performed with ATP treatment.

### P2Y2 receptor mediates activation of EGFR and ERK1/2

**Fig 2:**
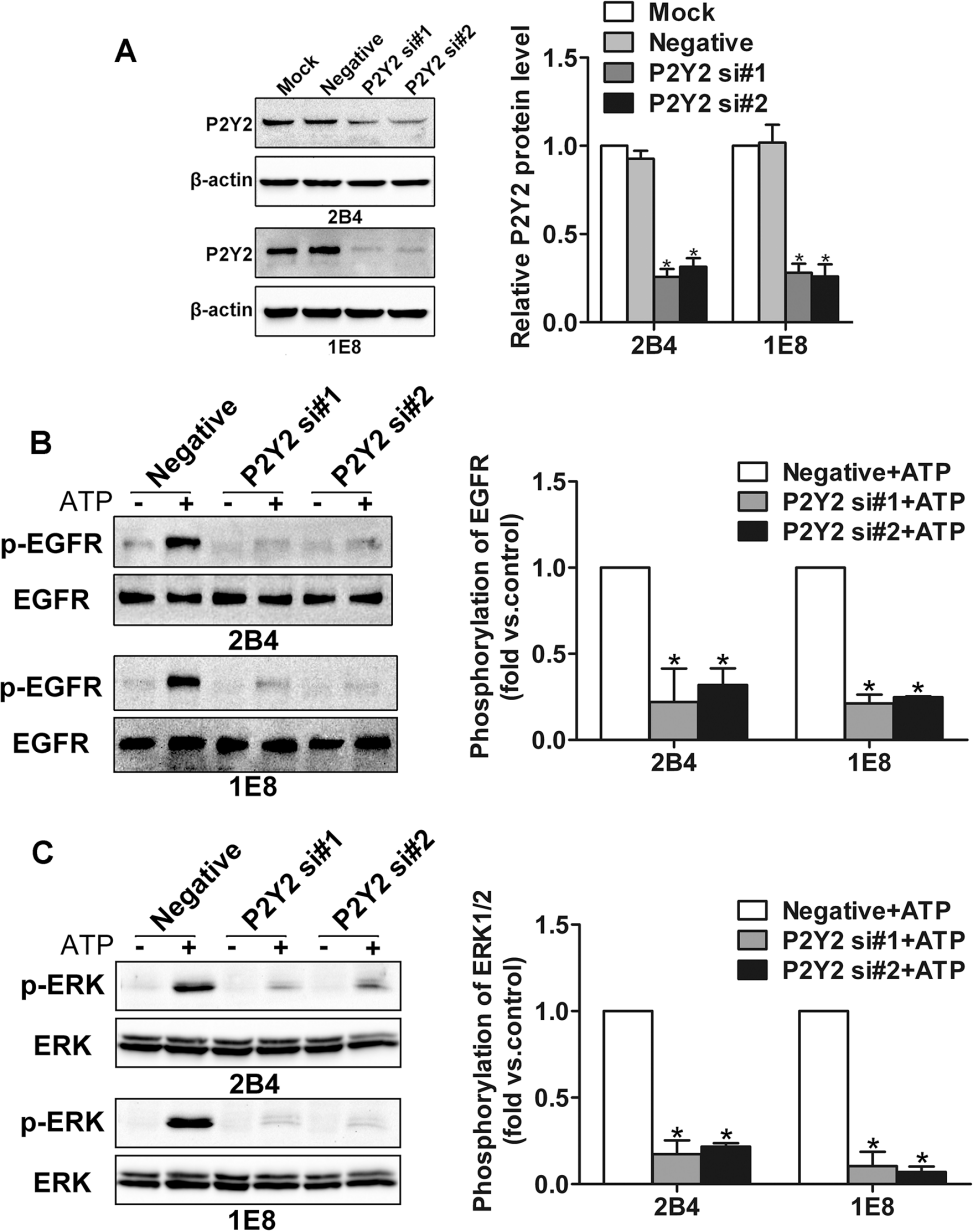
Knockdown of P2Y2 receptor inhibited ATP-induced activation of EGFR and ERK1/2. (**A**) 2B4 and 1E8 cells were transfected with two different P2Y2 siRNAs (P2Y2 si#1 and P2Y2 si#2) or a control siRNA (Negative), respectively. Knockdown efficiency was determined by western blotting. After transfection with two different P2Y2 siRNAs (P2Y2 si#1 and P2Y2 si#2) or a control siRNA (Negative) respectively, 2B4 and 1E8 cells were incubated with or without 100 μM ATP for 5 min. Then the protein was extracted for phosphorylation detection of (**B**) EGFR and (**C**) ERK1/2. Results were demonstrated by histograms to quantify the expression levels. Data were presented as means ± SD (vertical bars). Three independent experiments were performed. *p<0.05.

To identify the role of P2Y2 receptor in ATP-induced activation of EGFR and ERK1/2, siRNA was introduced to silence the expression of P2Y2 receptor in 2B4 and 1E8 cells. Prominent knockdown efficiency was identified by western blotting (Fig 2A). After incubation with 100 μM ATP for 5 min, the phosphorylation of EGFR and ERK1/2 was examined using western blotting. Here, we found that ATP treatment induced activation of EGFR and ERK1/2 in control siRNA cells. After knockdown of P2Y2 receptor, the activation of EGFR and ERK1/2 induced by ATP was significantly suppressed (Fig 2B and 2C). Together, these data suggest that the ATP-induced activation of EGFR and ERK1/2 was predominantly regulated by P2Y2 receptor.

### EGFR is involved in ATP-induced activation of ERK1/2

**Fig 3:**
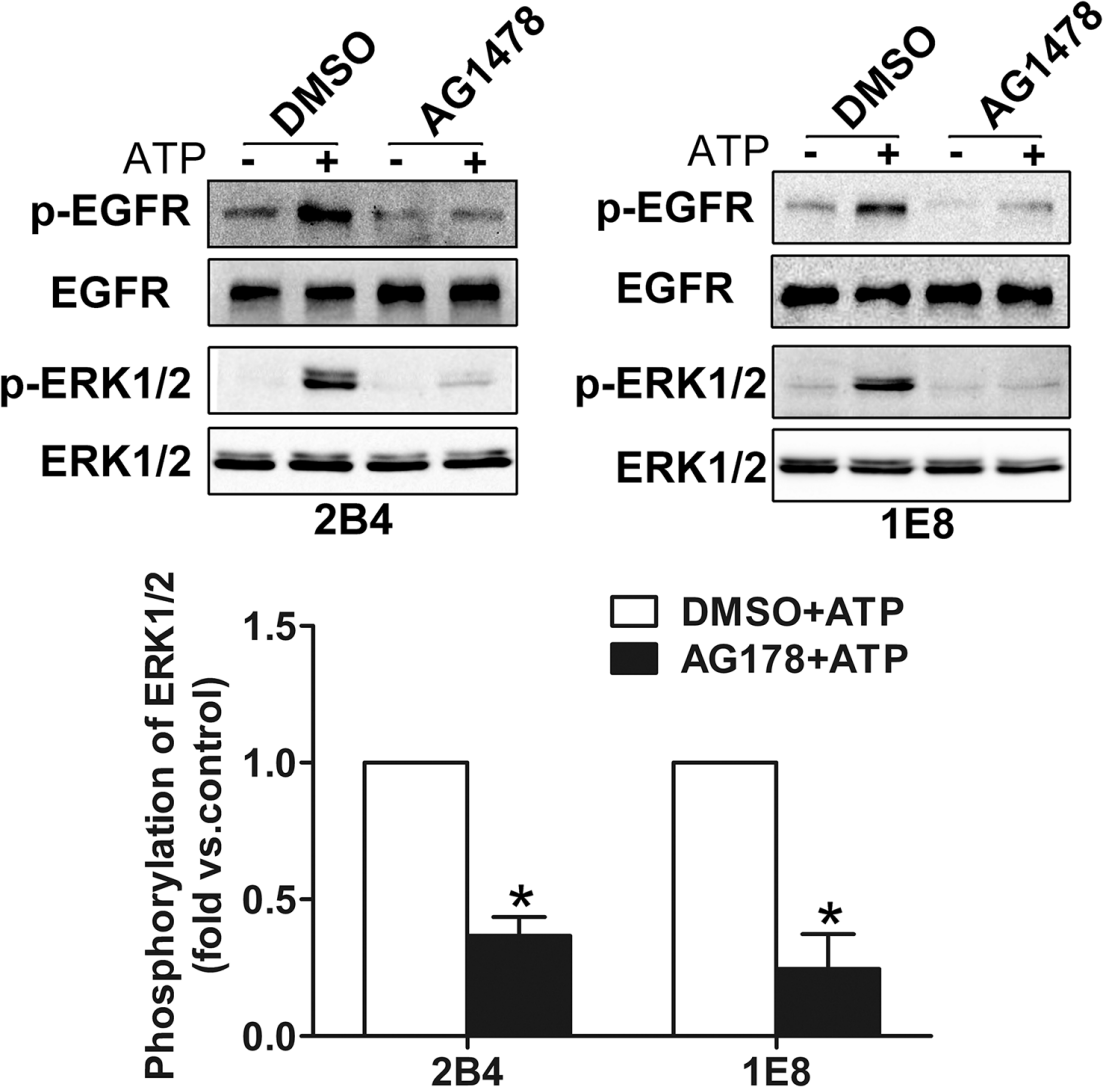
ATP-induced activation of ERK1/2 was dependent on EGFR activation. After incubation with 100 nM AG1478 (EGFR inhibitor) for 30 min, 2B4 and 1E8 cells were treated with 100 μM ATP for 5 min. Phosphorylation of EGFR and ERK1/2 was measured by western blotting. Results were demonstrated by histograms to quantify the expression levels. Data were presented as means ± SD (vertical bars). Three independent experiments were performed. *p<0.05.

To explore the role of EGFR in ATP-induced activation of ERK1/2, AG1478 (100 nM), a selective inhibitor of EGFR, was used before ATP treatment. Fig 3 demonstrated that ATP induced activation of EGFR and ERK1/2 in control group (DMSO-treated cells), whereas pretreatment with AG1478 significantly suppressed ATP-induced phosphorylation of EGFR and ERK1/2, suggesting that ATP activates ERK1/2 pathway through EGFR activation.

### P2Y2-EGFR-ERK1/2 pathway is involved in prostate cancer cell invasion and migration

**Fig 4:**
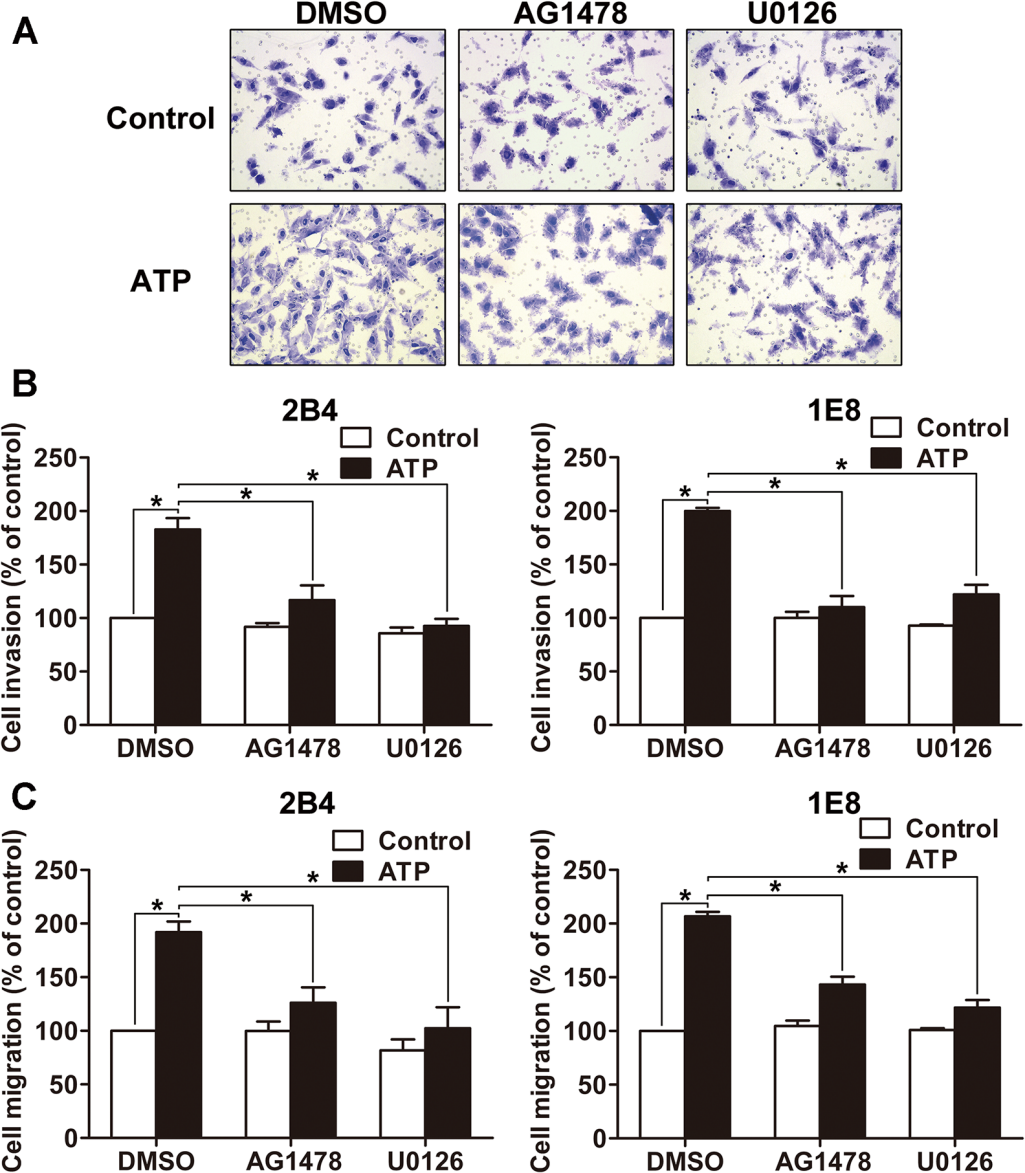
Effects of EGFR and ERK1/2 activation on ATP-mediated invasion and migration of prostate cancer cells. (**A**) Cells were harvested, resuspended and pretreated with AG1478, U0126 or DMSO for 30 min, respectively. Then cells were incubated in Transwell plate with or without 100 μM ATP for 12 h, invaded/migrated cells were stained with crystal violet and observed under a microscope at × 200 magnification. (**B**) Effects of AG1478 and U0126 on the invasion of 2B4 and 1E8 cells. (**C**) Effects of AG1478 and U0126 on the migration of 2B4 and 1E8 cells. Results were demonstrated by histograms, and data were presented as means ± SD (vertical bars). Three independent experiments were performed. *p<0.05.

Next, to investigate whether EGFR-ERK1/2 pathway mediated ATP-induced invasion and migration, we pretreated 2B4 and 1E8 cells with AG1478 (EGFR inhibitor, 100 nM) and U0126 (MEK1/2 inhibitor, 10 μM) respectively before 100 μM ATP stimulation. Invasion assay and migration assay revealed that ATP treatment enhanced cell invasion and migration in control group (DMSO-treated cells). But in AG1478- or U0126-treated groups, the ATP-promoted cell invasion and migration was inhibited (Fig 4A–4C). Since P2Y2 receptor predominantly mediates ATP-induced activation of EGFR and ERK1/2 as described above, and P2Y2 receptor is responsible for ATP-stimulated prostate cancer cell invasion and migration as demonstrated in our previous study [6], the results strongly suggest that P2Y2-EGFR-ERK1/2 pathway is involved in the regulation of prostate cancer cell invasion and migration.

### P2Y2-EGFR-ERK1/2 pathway contributes to IL-8 upregulation

**Fig 5:**
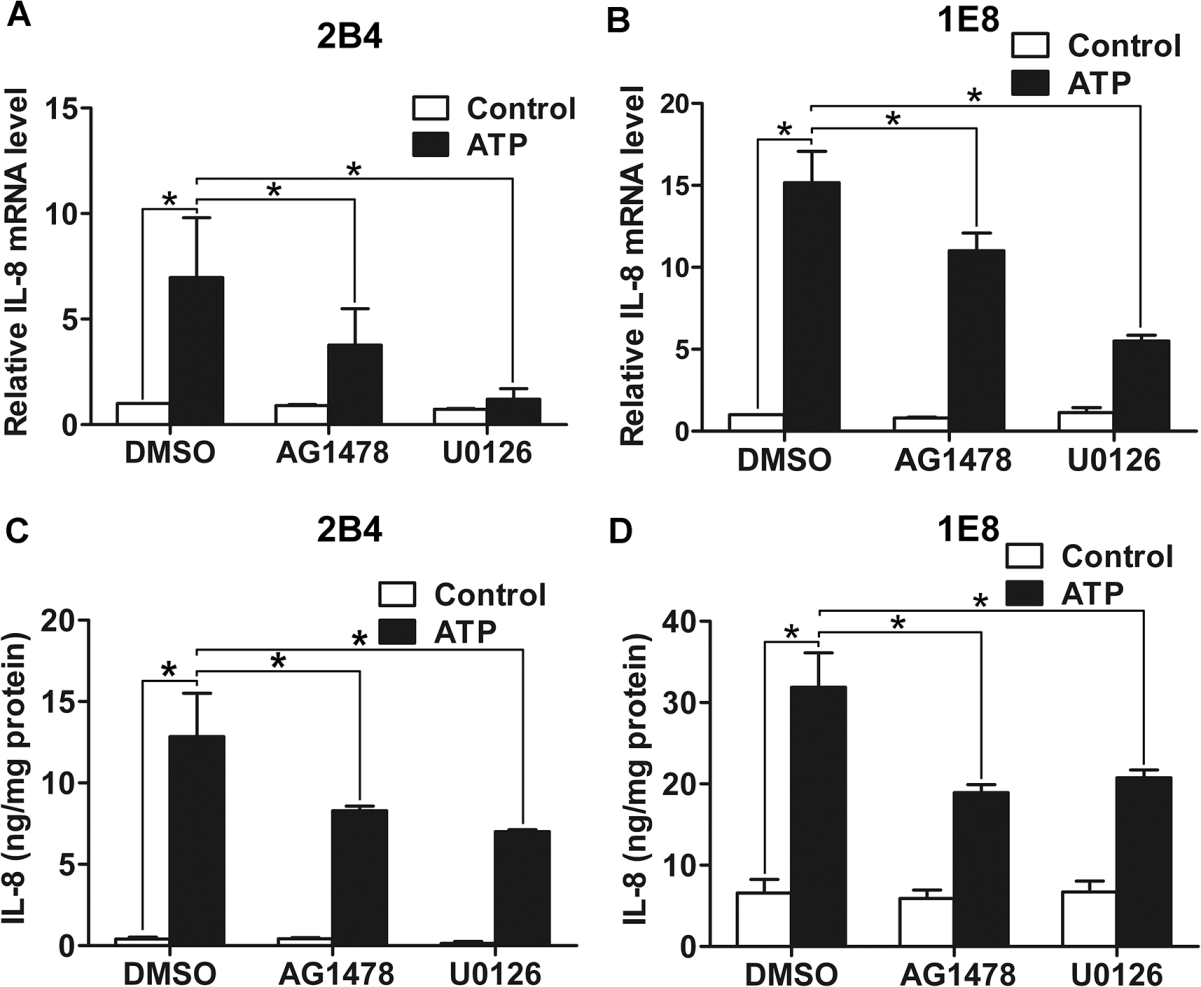
IL-8 production was increased after activation of EGFR and ERK1/2 by ATP. 2B4 and 1E8 cells were pretreated with AG1478, U0126 or DMSO for 30 min, respectively. Then cells were incubated with or without 100 μM ATP for 12 h. (**A**) and (**B**) mRNA level of IL-8 was detected by real-time PCR. (**C**) and (**D**) Protein level of IL-8 in cell supernatant was examined by ELISA assay. Results were demonstrated by histograms to quantify the expression levels. Data were presented as means ± SD (vertical bars). Three independent experiments were performed. *p<0.05.

Our previous study has shown that ATP and UTP both stimulated the production of IL-8, and knockdown of P2Y2 suppressed the secretion of IL-8 [6]. Here, 2B4 and 1E8 cells were incubated with 100 nM AG1478 (EGFR inhibitor) or 10 μM U0126 (MEK1/2 inhibitor) respectively before 100 μM ATP stimulation. Using real-time PCR and ELISA assay, we found that blockade of EGFR activation attenuated ATP-promoted IL-8 expression and secretion, while blockade of ERK1/2 activation also inhibited the expression and secretion of IL-8 (Fig 5A–5D). As ATP induced activation of EGFR and ERK1/2 via P2Y2 receptor, together with the previous results that silencing of P2Y2 with siRNA significantly suppressed ATP-promoted IL-8 production [6], these data strongly indicate that ATP promotes the expression and secretion of IL-8 via P2Y2-EGFR-ERK1/2 pathway.

### Role of IL-8 in ATP-promoted cell invasion and migration

**Fig 6:**
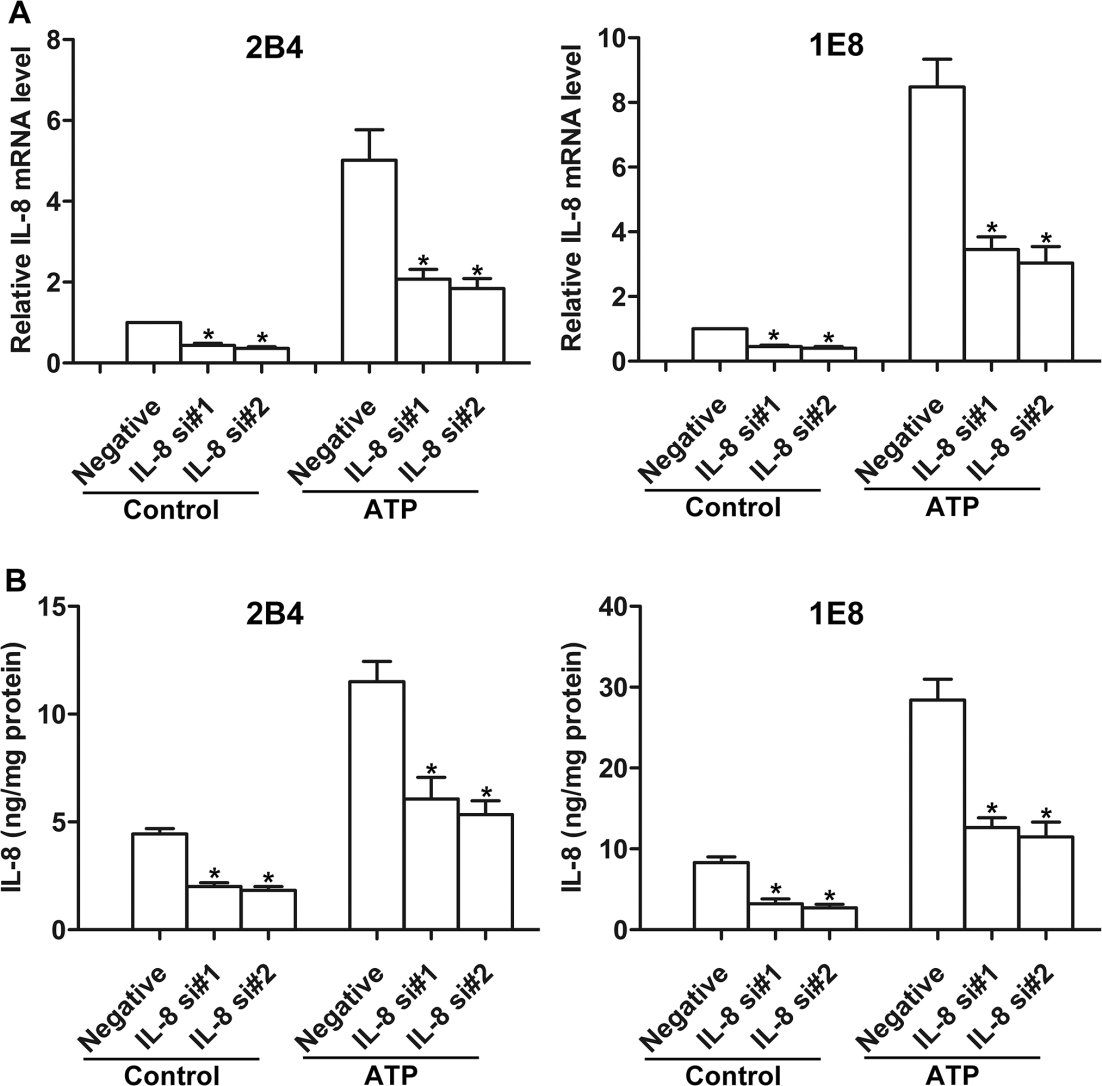
Effects of IL-8 siRNAs on ATP-mediated IL-8 production. 2B4 and 1E8 cells were transfected with two different IL-8 siRNAs (IL-8 si#1 and IL-8 si#2) or a control siRNA (Negative), respectively. Cells were treated with or without 100 μM ATP for 12 h. (**A**) IL-8 mRNA expression was detected by real-time PCR, and (**B**) IL-8 secretion was measured by ELISA assay. Results were demonstrated by histograms to quantify the expression levels. Data were presented as means ± SD (vertical bars). Three independent experiments were performed. *p<0.05 vs. Negative.

**Fig 7:**
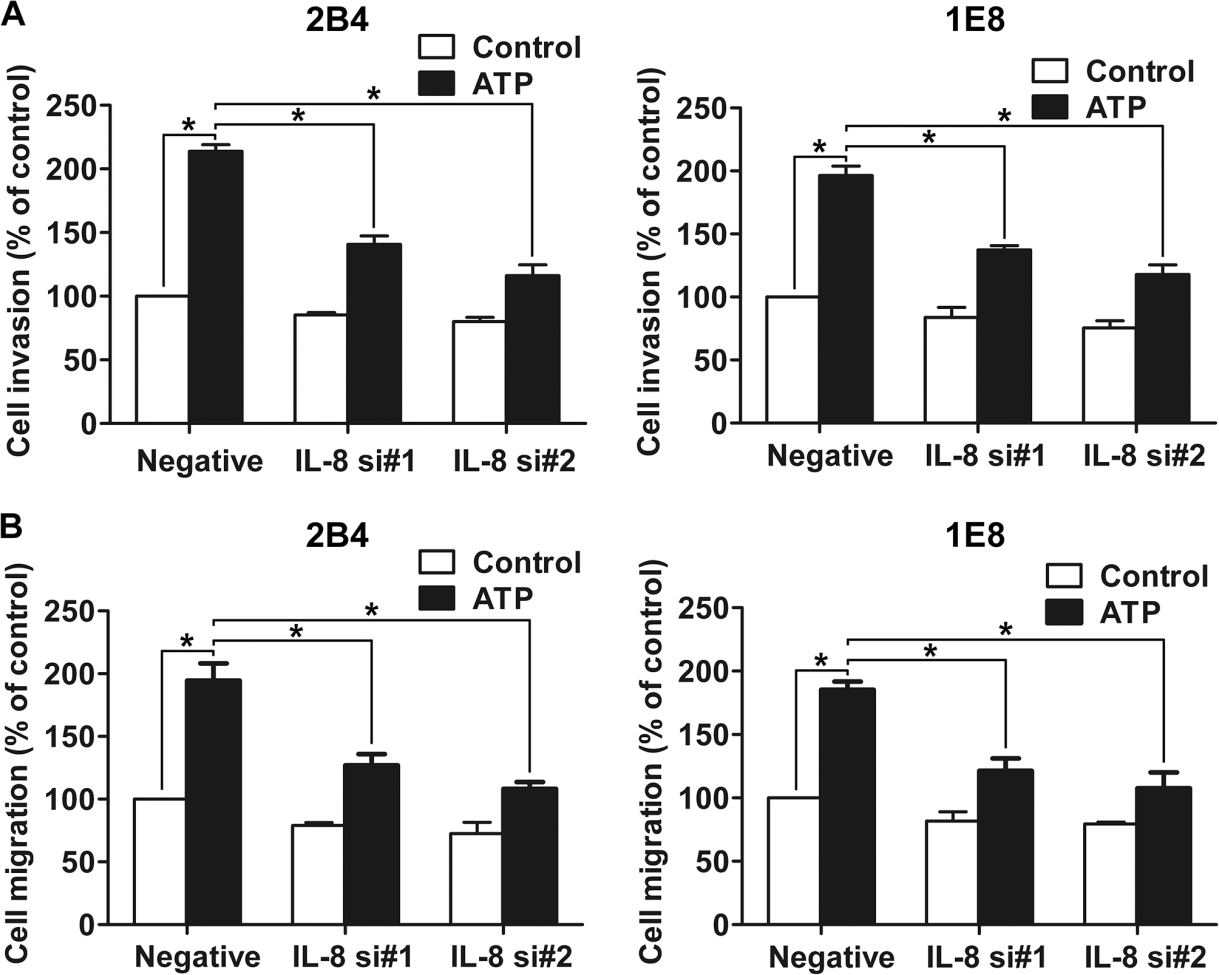
Knockdown of IL-8 attenuated ATP-mediated invasion and migration of prostate cancer cells. After knockdown of IL-8 by siNRA, cells were treated with or without 100 μM ATP, and then subjected to (**A**) invasion assay and (**B**) migration assay. Results were demonstrated by histograms, and data were presented as means ± SD (vertical bars). Three independent experiments were performed. *P<0.05.

Upregulation of IL-8 expression is associated with invasion and metastasis in prostate cancer [9]. Therefore, we silenced the expression of IL-8 in 2B4 and 1E8 cells with siRNA to examine the role of IL-8 in ATP-promoted invasion and migration. Two different IL-8 siRNAs were used, and real-time PCR and ELISA assay showed that the expression and secretion of IL-8 were suppressed after transfection with IL-8 siRNAs (Fig 6A and 6B). We also found that IL-8 siRNA treatment could suppress the ATP-mediated increase in IL-8 production (Fig 6A and 6B). Moreover, after knockdown of IL-8, ATP-stimulated cell invasion and migration of prostate cancer cells was greatly inhibited, suggesting that IL-8 is one of the important players in the regulation of ATP-promoted cell invasion and migration (Fig 7A and 7B)**.**

## Discussion

**Fig 8:**
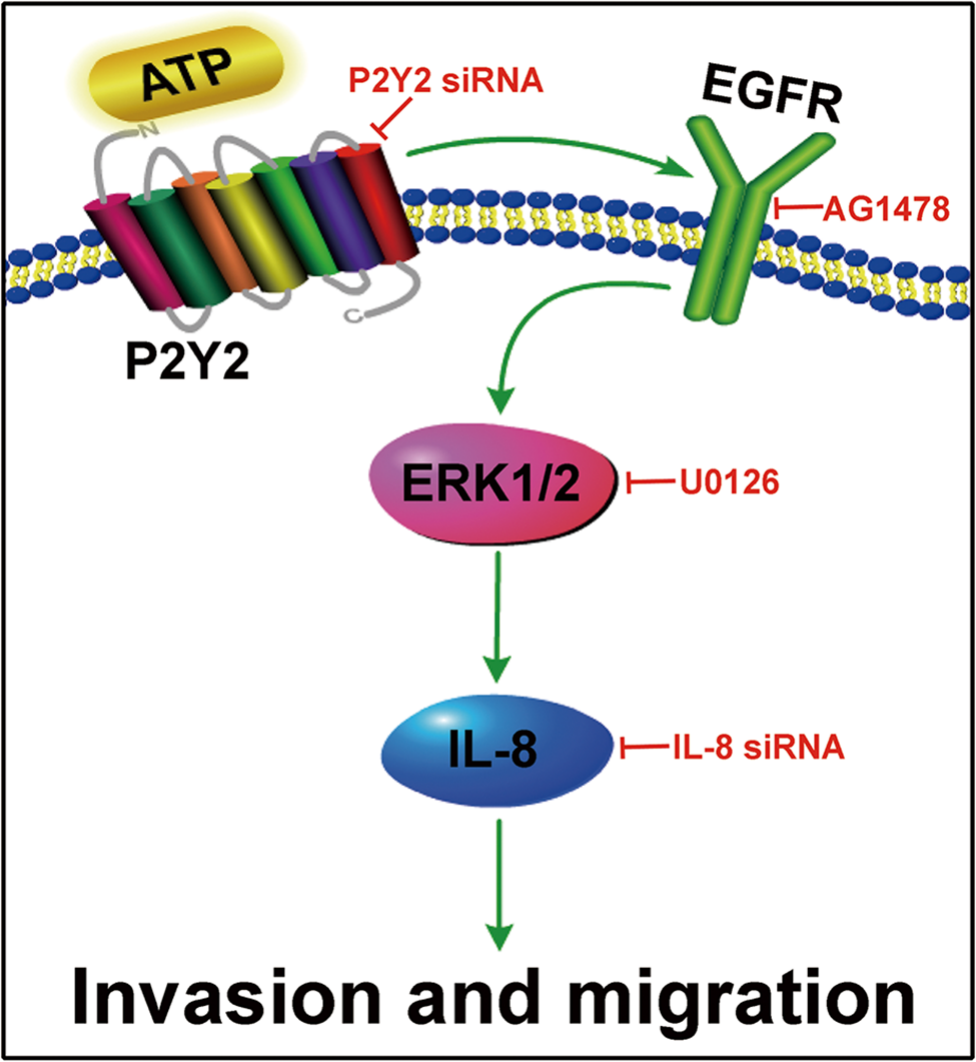
Diagram depicting the involvement of P2Y2-EGFR-ERK1/2 pathway and IL-8 upregulation in ATP-promoted invasion and migration of prostate cancer cells.

A number of studies have found that in tumor microenvironment extracellular ATP and its derivatives may act as detrimental signals in tumor progression [15, 16]. Our previous study showed that activation of P2Y2 by extracellular ATP promotes cell invasion and metastasis of prostate cancer cells *in vitro* and *in vivo* [6]. Belonging to G protein-coupled receptor (GPCR) subfamilies, P2Y2 receptors have been reported to couple with multiple intracellular signaling pathways [17, 18]. Most of these pathways are regulated simultaneously by EGFR activation and involved in tumor invasion and metastasis [19]. However, there is no convincing evidence so far that P2Y2 receptor promotes prostate cancer progression via EGFR. In this study, we revealed that EGFR and ERK1/2 could both be activated by ATP or UTP stimulation. Knockdown of P2Y2 receptor suppressed ATP-induced phosphorylation of EGFR and ERK1/2. In addition, blockade of EGFR suppressed ATP-regulated activation of ERK1/2. Further experiments showed that activation of EGFR-ERK1/2 pathway increased ATP-promoted IL-8 production, which then promoted the invasion and migration of prostate cancer cells. Together with our previous study that activation of P2Y2 receptor by ATP promotes cell invasion and metastasis of prostate cancer cells, this study strongly indicates that P2Y2 receptor promotes prostate cancer cell invasion and migration mainly via activation of EGFR-ERK1/2 pathway and upregulation of IL-8 production (Fig 8).

Multiple pathways have been identified downstream of GPCR. There are several possible explanations for the pro-invasive effect after P2Y2 receptor activation. One pathway is that ATP stimulates G_qα_-dependent phospholipase C (PLCb) activity, which generates inositol 1,4,5-trisphosphate (IP3) and diacylglycerol (DAG), resulting in an elevation in the intracellular calcium concentration and DAG-dependent activation of protein kinase C (PKC), respectively. PLCb2 has been shown to be important for breast cancer cell migration [20]. Changes in intracellular Ca^2+^ concentration alter essential processes in tumor cell migration and invasion [21]. PKC regulates metastasis through phosphorylation of many key proteins in different steps of metastasis. All of these regulatory elements are actively involved in our prostate cancer studies. In our previous studies, we observed remarkable accumulation of IP3 and significant intracellular Ca^2+^ mobilization in prostate cancer cells after ATP treatment [22]. This is obviously due to activation of PLCb by G-protein coupled P2Y2 receptor.

Another pathway is that the P2Y2 receptor can interact with αVβ_3/5_ integrins via an extracellularly oriented RGD domain to regulate the activities of G_12_-dependent Rho, Go-dependent Rac, LIM kinase, and cofilin, proteins that regulate actin cytoskeletal rearrangements which are important features of cell migration [23]. We previously demonstrated that ATP induced activation of Rac1 and Cdc42, promoted the formation of lamellipodia and filopodia, and increased the motility of prostate cancer cells [12], indicating a possible role of P2Y2 receptor in prostate cancer cell motility.

P2Y2 receptor may also interact with EGFR to activate ERK1/2 pathway since there are some evidences that transactivation of P2Y2 receptor and EGFR exists in some cell types, e.g., membrane distribution of the P2Y2 transregulated by EGFR in blood vessel smooth muscle cells [24], promotion of the formation of EGFR/ErbB3 heterodimers by P2Y2 receptor in salivary gland cells [25]. Phosphorylation of EFGR by P2Y2 receptor is thought mainly due to Src-dependent recruitment of P2Y2 receptor to a signaling complex containing EGFR in response to P2Y2 ligands [26]. In the present study, we revealed that EGFR and ERK1/2 could be activated by extracellular ATP or UTP stimulation. Knockdown of P2Y2 receptor suppressed ATP-induced phosphorylation of EGFR and ERK1/2. To our knowledge, our present data represent the first example that EGFR participates in P2Y2 receptor-regulated cell invasion of prostate cancer.

It is well known that EGFR participates in diverse cellular processes such as growth, differentiation, tumorigenesis, invasion and metastasis of cancer [27, 28]. Increased EGFR expression is often detected in prostate cancer, and is associated with poor prognosis [29]. Monoclonal antibody against EGFR has gained an effective improvement in patients with prostate cancer [30]. As one subfamily of MAPKs, ERK1/2 is the best characterized cytoplasmic kinase activated by EGFR, and inhibition of ERK1/2 has been utilized as a biomarker for EGFR inhibitor action [31]. Inhibition of ERK1/2 pathway also has been considered as an important treatment strategy for prostate cancer [32, 33]. In this study, we demonstrated a significant function of P2Y2-EGFR-ERK1/2 pathway in mediating prostate cancer cell invasion and migration. Together with our previous observation that extracellular ATP is an important pro-invasive agent within tumor microenvironment, this may help to elucidate the regulating mechanism(s) underlying EGFR activity in prostate cancer progression.

IL-8 has been found to be highly expressed in androgen-independent metastatic cell lines, such as PC-3 cell line [9]. Clinical studies also reveal that IL-8 production is elevated in tumor tissue and serum of patients with prostate cancer, and there is a direct correlation between high level of IL-8 and tumor progression [34]. Increase of IL-8 production is associated with tumor angiogenesis, proliferation and metastasis of prostate cancer *in vitro* and *in vivo* [35]. Here, our study proves that ATP upregulated the expression and secretion of IL-8 via P2Y2-EGFR-ERK1/2 pathway, and IL-8 production contributed to ATP-promoted invasion and migration of prostate cancer cells.

In summary, our results demonstrate that P2Y2 receptor upregulates IL-8 production, thereby promoting the invasion and migration of prostate cancer cells. Cross-talking with EGFR and subsequently activation of ERK1/2 pathway may be one of the important mechanisms of P2Y2 receptor function. Therefore, therapies that target P2Y2-EGFR-ERK1/2 pathway may provide effective treatment strategies for prostate cancer.
